# Supplementary material for: From sequence to enzyme mechanism using multi-label machine learning
Source: BMC Bioinformatics. 2014 May 19;15:150. doi: 10.1186/1471-2105-15-150 (PMC4229970; doi:10.1186/1471-2105-15-150)
Supplement: Additional file 2 — Java code of ml2db. Additional file ml2db_code.tar.gz contains the Java source code to run the multi-label machine learning experiments and save the results to database. The code’s Javadoc is included. [file 1471-2105-15-150-S2.zip › additional file 2/ml2db/utils/doc/uk/ac/standrews/utils/main/diff/Difference.html]

Difference


JavaScript is disabled on your browser.


- Overview
- Package
- Class
- Use
- Tree
- Deprecated
- Index
- Help

- Prev Class
- Next Class

- Frames
- No Frames

- All Classes

- Summary:
- Nested |
- Field |
- Constr |
- Method

- Detail:
- Field |
- Constr |
- Method


uk.ac.standrews.utils.main.diff

## Class Difference

- java.lang.Object
- - uk.ac.standrews.utils.main.diff.Difference

- ---

    

  ```
  public class Difference
  extends java.lang.Object
  ```

  Represents a difference, as used in `Diff`. A difference consists
  of two pairs of starting and ending points, each pair representing either the
  "from" or the "to" collection passed to `Diff`. If an ending point
  is -1, then the difference was either a deletion or an addition. For example,
  if `getDeletedEnd()` returns -1, then the difference represents an
  addition.

- - ### Field Summary

    Fields

    | Modifier and Type | Field and Description |
    | --- | --- |
    | `static int` | `NONE` |
  - ### Constructor Summary

    Constructors

    Constructor and Description

    `Difference(int delStart,
    int delEnd,
    int addStart,
    int addEnd)`

    Creates the difference for the given start and end points for the
    deletion and addition.
  - ### Method Summary

    Methods

    | Modifier and Type | Method and Description |
    | --- | --- |
    | `boolean` | `equals(java.lang.Object obj)` Compares this object to the other for equality. |
    | `int` | `getAddedEnd()` The point at which the addition ends, if any. |
    | `int` | `getAddedStart()` The point at which the addition starts, if any. |
    | `int` | `getDeletedEnd()` The point at which the deletion ends, if any. |
    | `int` | `getDeletedStart()` The point at which the deletion starts, if any. |
    | `void` | `setAdded(int line)` Sets the point as added. |
    | `void` | `setDeleted(int line)` Sets the point as deleted. |
    | `java.lang.String` | `toString()` Returns a string representation of this difference. |

    - ### Methods inherited from class java.lang.Object

      `getClass, hashCode, notify, notifyAll, wait, wait, wait`

- - ### Field Detail


    - #### NONE

      ```
      public static final int NONE
      ```

      See Also:
      :   Constant Field Values
  - ### Constructor Detail


    - #### Difference

      ```
      public Difference(int delStart,
                int delEnd,
                int addStart,
                int addEnd)
      ```

      Creates the difference for the given start and end points for the
      deletion and addition.
  - ### Method Detail


    - #### equals

      ```
      public boolean equals(java.lang.Object obj)
      ```

      Compares this object to the other for equality. Both objects must be of
      type Difference, with the same starting and ending points.

      **Overrides:**
      :   `equals` in class `java.lang.Object`


    - #### getAddedEnd

      ```
      public int getAddedEnd()
      ```

      The point at which the addition ends, if any. A value equal to
      `NONE` means this must be an addition.


    - #### getAddedStart

      ```
      public int getAddedStart()
      ```

      The point at which the addition starts, if any. A value equal to
      `NONE` means this must be an addition.


    - #### getDeletedEnd

      ```
      public int getDeletedEnd()
      ```

      The point at which the deletion ends, if any. A value equal to
      `NONE` means this is an addition.


    - #### getDeletedStart

      ```
      public int getDeletedStart()
      ```

      The point at which the deletion starts, if any. A value equal to
      `NONE` means this is an addition.


    - #### setAdded

      ```
      public void setAdded(int line)
      ```

      Sets the point as added. The start and end points will be modified to
      include the given line.


    - #### setDeleted

      ```
      public void setDeleted(int line)
      ```

      Sets the point as deleted. The start and end points will be modified to
      include the given line.


    - #### toString

      ```
      public java.lang.String toString()
      ```

      Returns a string representation of this difference.

      **Overrides:**
      :   `toString` in class `java.lang.Object`


- Overview
- Package
- Class
- Use
- Tree
- Deprecated
- Index
- Help

- Prev Class
- Next Class

- Frames
- No Frames

- All Classes

- Summary:
- Nested |
- Field |
- Constr |
- Method

- Detail:
- Field |
- Constr |
- Method
